# GP survivors’ experiences working with family violence survivors: a qualitative study

**DOI:** 10.3399/BJGP.2026.0032

**Published:** 2026-06-30

**Authors:** Jennifer Neil, Clare Delany, Elizabeth McLindon, Kelsey Hegarty

**Affiliations:** 1 Department of General Practice and Primary Care, The University of Melbourne, Melbourne, VIC, Australia; 2 Monash University School of Medicine, Monash University, Clayton, VIC, Australia; 3 Department of Medical Education, The University of Melbourne, Melbourne, VIC, Australia; 4 The Royal Women’s Hospital, Parkville, VIC, Australia

**Keywords:** doctor wellbeing, domestic violence, family practice, family violence, general practice, primary health care

## Abstract

**Background:**

Domestic and family violence (DFV) is common worldwide and GPs are not immune. DFV survivors frequently present to primary care where GP DFV survivors often encounter them. Little literature and no qualitative studies, to the authors’ knowledge, have looked at the emotional and professional effects that these encounters have on survivor GPs, including how it affects their ability to care for survivor patients.

**Aim:**

To answer the question: what are the experiences of survivor GPs when working with patient survivors of DFV?

**Design and setting:**

A phenomenological qualitative study, to gain a deep understanding of Australian GP DFV survivors’ experiences when working with survivor patients.

**Method:**

Participants were Australian GPs self-identifying as survivors of all forms of DFV. Participants were recruited from two Australian GP online groups using purposive sampling. Semi-structured interviews took place and were audio-recorded and transcribed verbatim. Reflexive thematic analysis was undertaken.

**Results:**

Participants (*n* = 20) were women and worked in a range of locations throughout Australia. Four themes were identified: ‘my experiences can be my superpower’ (encompassing the subthemes: ‘having a sixth sense’ and ‘going above and beyond’); ‘connecting with survivors’ stories’; ‘wearing a cloak’; and ‘my experiences give me a sense of purpose’.

**Conclusion:**

Although GP DFV survivors commonly experienced difficult emotions when working with survivor patients they had a commitment to this work that stemmed from personal DFV experiences. This increased their empathy for survivors and gave them a deep understanding of their circumstances, allowing them to work powerfully with survivors, aiming to improve their situation.

## How this fits in

Domestic and family violence (DFV) is common and GPs are not immune; however, we know little about the emotional and professional effects on survivor GPs when caring for survivor patients. This is the first qualitative study, to the authors’ knowledge, to focus on GP DFV survivors’ experiences when working with DFV survivor patients. This study found that although GP survivors commonly experience difficult emotions, they have a commitment to their survivor patients that stems from their own experiences of DFV, an increase in empathy, and an increased understanding of their survivor patient’s situations that allowed them to powerfully work with DFV survivors in their clinics. This has implications for GP wellbeing and further study into how best to support GP survivors is recommended.

## Introduction

Domestic and family violence (DFV) is common and has significant mental and physical health effects on survivors.^1–3^ DFV is any behaviour within a domestic, family, or intimate relationship that causes physical, psychological, or sexual harm and includes intimate partner violence (IPV), child abuse, sibling abuse, and abuse by in-laws.^1,4^ DFV is common worldwide with one in three women being subjected to IPV and one in two children being subjected to child abuse.^2,3^ IPV tends to be a gendered issue with women more commonly survivors of violence from their male partner.^2,5^

Doctors are not immune to DFV;^6,7^ a 2022 systematic review (1989–2021) of 51 articles involving 25 111 health professionals found lifetime prevalence of DFV was 41.8% of women and 14.8% of men.^6^ Overall, doctors had a lower prevalence rate than other health professional groups (12.1%), but faced stigma when disclosing DFV, which was associated with underreporting.^6,7^ The literature suggests that a personal experience of DFV is common among GPs (family physicians) with prevalence ranging from 12% to 33.6%.^8–11^ Survivor patients are more likely to attend their GP for support compared with other professionals, so survivor GPs will commonly encounter survivor patients.^12^ The terminology ‘survivor’ is used for both patients and GPs who have been subjected to DFV as this terminology is strengths-based.

Studies have shown that clinician DFV survivors respond in varied ways to survivor patients.^6^ Some studies found survivor clinicians more likely to identify survivor patients and demonstrated increased empathy towards them, whereas other studies indicated no difference in care.^6,13^ An Australian study of hospital clinicians found that DFV survivors were doing ‘more of the work’ with survivors and offered better clinical care.^13^ Of the scant literature specific to doctors’ own DFV experiences, inconsistency characterises the findings. Four studies (one qualitative, three quantitative) showed increased confidence to manage survivor patients and/or increased empathy.^8,10,14,15^ In contrast, quantitative studies showed that survivor doctors were no more likely to identify survivor patients^16^ and that doctor survivors of child abuse had negative attitudes towards survivors of IPV.^17^ With regard to studies specifically on survivor GPs working with survivors there have been two quantitative studies^8,10^ and no qualitative studies to the authors’ knowledge.

To fill this gap in the literature, there is a need to explore in depth the effect of being a survivor GP on their clinical practice. General practice (family medicine) is a unique healthcare setting marked by continuity of care and long-term relationships; with survivor GPs likely to see survivor patients frequently.^12,18^

Little is known about the emotional and professional effects on survivor GPs of caring for survivor patients. This study aims to address this gap by exploring the impact on GP DFV survivors of caring for patient DFV survivors, and how GP survivors respond to survivor patients. This study asked the question: what are the experiences of survivor GPs when working with patient survivors of DFV?

## Method

### Study design

We used a phenomenological qualitative approach to interviewing to encourage GP participants to talk about how they frame and respond to the intersection between being a survivor GP and working with survivor patients.^19^ The research paradigm of phenomenology was used because it explores the essence of lived experiences and this overarching approach guided both data collection and analysis.^19^ Interviews, conducted by the first author, an experienced GP and DFV educator, were supported by three experienced researchers (the senior author, a GP; the third author, a DFV social worker; and the second author, an ethicist).

### Participants and sampling

Participants were GPs who had worked in general practice in Australia who self-identified as having experienced any form of DFV. Participants were recruited using purposive sampling from two Australian GP Facebook pages, one open to GPs of any gender in Australia and one specifically for GP mothers. We interviewed 20 GPs, and a A$100 e-gift card was provided to participants. We were guided by the principle of information power that informed our sample size.^20^ Our sample was diverse and contained a group of participants who met the aims of the study that included self-identifying as being a survivor of DFV.

### Data collection

Drawing from previous literature, we developed and refined (through pilot testing) a semi-structured interview guide to gain a deep understanding of participants’ interactions with survivor patients. The questions (Supplementary Box S1) explored how GPs identify and respond to survivor patients and their families, supports that were utilised, and the workplace role. This paper focuses on working with survivor patients. A brief demographic pre-survey was developed (Supplementary Box S2).

Written consent was obtained and 20 interviews were conducted between April and June 2025; 19 via Zoom for geographical reasons and one interview in person. Interviews were an average of 50 min (range 29–71 min). Several participants experienced emotions as they recalled patient stories and the first author was able to respond effectively as per the distress protocol. None of the participants wished to conclude the interview early. Data collection and analysis occurred iteratively allowing for deep engagement with the data.^21^ Reflexive journalling and discussions occurred throughout data collection and analysis.^22^

### Data analysis

Audio-recordings were transcribed verbatim with the first two recordings transcribed by the first author and the rest by a professional transcription service. A reflexive thematic analysis approach was used as the six phases of analysis ensured that the thoughts and experiences of GPs were rigorously captured, categorised, and organised as recurring themes in the data through an inductive approach (Box 1).^23^

**Box 1.** Steps of analysis^23^

Step	Action
**Data familiarisation**	The first author listened to all interviews, read and re-read transcripts
**Coding**	The first author coded the de-identified transcripts using NVivo (version 15, Lumivero). The other three authors reviewed and refined the coding tree
**Generating initial themes**	Initial themes were constructed and reviewed by all authors
**Developing and reviewing themes**	The first author reviewed and redeveloped themes while also recoding the dataset. This was reviewed by the other three authors
**Refining, defining, and naming themes**	The analysis was fine-tuned and themes were named by all authors
**Writing up**	The first author completed the initial draft; all authors were involved in reviewing and refining

## Results

Participants (*n* = 20) were all women, mostly working in metro locations across Australia (see Table 1). Most (*n* = 18) participants were experienced GPs, and two were GP trainees. Their experiences of DFV varied from childhood to adulthood. Most (*n* = 16) reported frequent contact with DFV survivor patients. All participants had named their experience as DFV when entering the study and all had taken action around this issue, although participants were not specifically asked whether the violence continued or not.

**Table 1.** Demographics

Characteristic	Participants, *n*
**Age, years**	
31–35	3
36–40	5
41–45	6
46–50	5
51–55	1
**Length of time working in general practice, years**	
<5	3
5–10	3
10–15	10
15–20	2
>20	2
**State of Australia**	
Australian Capital Territory	3
New South Wales	6
Northern Territory	0
Queensland	4
Southern Australia	0
Tasmania	1
Victoria	5
Western Australia	1
**Location of practice^a^**	
Metropolitan	12
Outer metropolitan	4
Regional	4
Rural	1
**How many hours of DFV training**	
0–2	7
3–8	3
9–14	5
15–20	3
>20	2
**How often sees DFV survivors**	
Every day	9
2–3 times a week	5
Weekly	2
Once a month	3
Once every 6 months	1
**Type of abuse experienced^b^**	
Child abuse	14
Sibling abuse	1
Abused by a parent as an adult	2
IPV	11
In-laws	1

^a^One participant works in both metropolitan and rural. Metropolitan areas are major cities; regional is a populated area, town, small city but not a major capital city. ^b^Many participants experienced more than one type. DPV = domestic and family violence. IPV = intimate partner violence.

### Themes

Four themes were identified from the data: ‘my experiences can be my superpower’ (subthemes: ‘having a sixth sense’ and ‘going above and beyond’); ‘connecting with survivors’ stories’; ‘wearing a cloak’; and ‘my experiences give me purpose’.

### Theme 1: my experiences can be my superpower

Most participants felt that they used the special knowledge and understanding they had gained through personal experience to be better doctors, well-equipped to support survivors. They perceived that they identified many survivors using their ‘sixth sense’ and had unique skills to respond to survivors, going ‘above and beyond’ for them. As one participant said:

*‘All of my patients who have disclosed family violence have been seen by other doctors – other doctors who didn’t see it before … the feedback from patients has consistently been that it’s really great to see a GP … who actually sees that dimension of themselves. I think it’s an absolute superpower that I’ve had this experience. It has increased my power as a healer so much.’* (P01, child abuse [CA] and IPV)

#### Having a sixth sense

All participants felt able to pick up subtle signs of abuse that they said stemmed from a deep intuition. They used descriptive language to outline this process using words such as, ‘sixth sense’, ‘spidey senses’, ‘a radar’, ‘gut feel’, ‘vibes’, ‘heebie-jeebies’, ‘antennas’, ‘familiarity alarm bell’. As one participant said, ‘I can smell it.’ (P17, CA and IPV). Participants discussed being able to identify abuse patterns, especially coercive control, because they were familiar with it from personal experience:

*‘You have this sixth sense that something’s not right … I guess because that sixth sense has served me well to help me survive, I just know not to ignore it when I’m in a consult.’* (P04, CA and abuse by parent as adult)

*‘There’s certainly sometimes little situations that give you the vibes, heebie-jeebies … when you're talking to somebody and something just doesn't quite feel right.’* (P16, CA)

Participants believed that they were more comfortable asking about DFV compared with their GP peers, which contributed to increased disclosures:

*‘So, I guess it* [being a survivor] *helps my work because I'll ask anyone anything.’* (P14, CA)

Overall, participants felt that they had a unique understanding of their survivor patients compared with GPs who were not survivors, which they felt underpinned stronger patient care:

*‘I do think that survivors themselves can cater to other survivors in ways that people who haven't had that experience can’t.’* (P15, CA and IPV)

*‘I’m sure I’m a better doctor as a result of all of this, unfortunately. Fortunately, but unfortunately. I’m sure I’m a better doctor.’* (P18, IPV)

Participants perceived that they were not only identifying many survivors, but they also felt that they were better doctors because of their experiences, going above and beyond for survivor patients (see Box 2).

Box 2. **Case story**

One participant saw a couple in her clinic and the women needed a cervical screening test (CST). The husband decided to stay in the room for the CST. ‘Something about it just felt weird.’ (P11, CA). So she wrote ‘do you need help’ on a piece of paper and left it with a pen behind the curtain where the patient was undressing. The patient wrote ‘get the police, he’s going to kill me’. The police were called and were waiting at the end of the consultation, arresting him and removing his access to a weapon. Describing the experience, the participant said: ‘You kind of think, I don't know, you’ve got to listen to those little bits of intuition, don't you, where you just know something’s not right?’ (P11, CA)

#### Going above and beyond

Most participants reported that they would go above and beyond for survivor patients out of a sense of solidarity and identification. This included sharing validation, creative solutions, and advice for survivor patients:

*‘I’d go above and beyond. I’d have superhuman energy to make sure they were protected and that they were treated well.’* (P01, CA and IPV)

*‘I think I’m probably GP extra to that child.’* (P14, CA)

Many participants wanted to give survivors the experience they themselves had never had with doctors.

*‘I’m being the person I wish I’d had access to.’* (P13, CA)

Participants felt the importance of validating survivors, because they themselves wished to be validated.

*‘I believe them. Some of the stories are so outrageous. But when you have been in outrageous situations yourself, you know that these things happen and are true.’* (P09, CA and IPV)

Participants also used their own experiences to inform creative problem solving and advice, and an understanding of supports and services:

*‘I’ve come up with some very creative ideas as well on problem solving because I’ve also had to be really creative about my own difficulties as well.’* (P02, IPV)

*‘Survivors themselves are aware of all the resources more than someone who hasn’t gone through it… you have a lot more nuanced information.’* (P15, CA and IPV)

Participants perceived they were better doctors and well-equipped to identify and support survivors due to their own experiences despite difficult emotions that could arise from connecting with survivor stories.

### Theme 2: connecting with survivors’ stories

Most participants strongly identified with survivor patients, especially but not exclusively when their stories had resonance with their own. This could lead to difficult emotions as well as empathy:

*‘They start to talk to me about different patterns of things like control, and gaslighting … there’s so much that is relatable in those stories.’* (P08, IPV)

Hearing survivor stories could lead to an emotional response, with participants describing encounters as ‘triggering’, ‘upsetting’, ‘tough’, ‘difficult’, and ‘distressing’:

*‘Anything… similar to what I went through is always going to trigger me. Because for me, there was a physical aspect. Anybody who has a physical aspect, then I immediately get that* [inhales], *a bit of an, ugh, I know what that feels like.’* (P05, In-law abuse)

*‘There were a lot of parallels in the story … I was like, wow, there’s a lot of similar stuff there … It was certainly distressing afterwards … That stuff never goes away.’* (P11, CA)

However, in general, participants felt they had more empathy that enabled them to figuratively walk in their survivor patient’s shoes:

*‘I think when you actually live through it, you actually understand those concepts better … it kind of gives you an accelerated way of empathising I think.’* (P03, CA)

*‘I feel like it makes me more empathetic because I feel like I can be in their shoes a little bit more ... it just makes me more empathetic and more wanting to help them.’* (P13, CA)

However, although feeling empathy, several participants felt frustrated when they perceived survivor patients to be ‘stuck’. These participants reflected that they themselves had moved beyond ‘stuck’ and could see that life was better on the other side:

*‘I really struggle … when they’re* [survivor patient] *into that contemplative or even pre-contemplative stage… I struggle also feeling angry at them for going back. It’s like I escaped. Why can’t you? Why can't you see?’* (P04, CA and abuse by parent as adult)

As participants identified with DFV survivors, they experienced an emotional response both in themselves and towards their survivor patients. This then led to a tension in their professional role including facing dilemmas about reciprocal sharing of their own story.

### Theme 3: wearing a cloak

Participants described experiencing tension in their consultations with survivors as they balanced staying professional with the emotions they were experiencing. Many participants had a ‘get on with it’ mentality described as ‘wearing a cloak’ as they put on their professional identity to shield them from being overwhelmed by emotion in the consultation:

*‘I think you put on a different cloak. I think it'll be okay, but … I've actually booked out the appointment after it … as a breather.’* (P11, CA)

Many participants used a cloak of professionalism to ensure that patients did not recognise that they had been emotionally affected or ‘triggered’ during the consultation because of concerns that it may negatively affect the patient:

*‘Sometimes I’m really glad I still wear a surgical mask so they can’t see my face … I just have to really hold myself together and go, okay, I understand … I can’t show it because I don’t want to upset her … So, it’s quite frustrating and I kind of have to let it all out when the patient’s not in the room.’* (P04, CA and abuse by parent as adult)

*‘It doesn't trigger anything. When you're in your professional role, you're in your professional role, you know?’* (P07, CA and abuse by parent as adult)

Participants reported trying to remain professional during the consultation and pushing through the emotions but would then use different coping mechanisms such as breaking into tears afterwards, needing a breather, booking out the next appointment or having a debrief:

*‘So, then it becomes a little awkward because you kind of go, I need to push that and I need to do the professional, and I will cry later.’* (P05, In-law abuse)

One participant described the process of how she maintained a professional demeanour to avoid the patient seeing that the consultation had upset her and her strategy for reducing any negative effects on patient care:

*‘It’s hard during a consult to do very much because the patient’s right there, so I try and compartmentalise it in that moment, to kind of get through the consult the best I can. Then afterwards I take a couple of minutes before bringing the next patient in, do some breathing exercises, I might write something down. I think journalling’s quite helpful. Then, if I felt that there was something that I missed telling the patient or something that could have helped them … I … call them later, when I’m feeling a bit better and say, there’s this other resource that I thought would be helpful for you … I don’t think that the patient has ever recognised that I was triggered.’* (P15, CA and IPV)

Participants also described there was tension in the consultation about the appropriateness of expressing emotions and crying in front of survivor patients. Participants who put on the cloak of professionalism held it together through the consultation before expressing their emotions later:

*‘I have to be professional and deal with my anxiety later and just focus on her.’* (P04, CA and abuse by parent as adult)

Conversely, some participants cried with survivor patients during the consultation. They acknowledged the risk of reducing focus on the patient or making things awkward, especially if there was not already a strong doctor–patient relationship. However, one participant felt that it showed the survivor patient that they understood their situation:

*‘Often, I won't cry but there have been times when I have cried a few tears because I am so sad about the situation … and I will cry in front of the patient because I'm human. I'm not a robot. I don’t apologise for it, apart from if it derails the conversation, which hopefully it doesn’t. It’s more to show that I really do get it.’* (P17, CA and IPV)

*‘The incident that happened to her was … similar to the incident that happened to me … I sat there, held her hand and I'm like, oh sorry, I'm going to cry on you for a minute ... She was like, are you all right? … because I know her very well, it helps. But occasionally, when there are people obviously you don’t know very well, you can’t really do that. So, then it becomes a little awkward.’* (P05, In-law abuse)

Although many participants did not disclose their own stories to patients, some disclosed parts of their stories to show that healing can occur after DFV. Participants spoke of a tension with balancing disclosure and keeping the focus on their patient:

*‘I have actually shared little bits of my history to say that I understand. I understand what domestic violence looks like when you're raising children … You have to do the work and that’s how you'll get stronger and not make those same mistakes.’* (P17, CA and IPV)

Despite feeling difficult emotions and having to continuously put on the cloak of professionalism, many participants felt a sense of purpose in their work with survivors because of their own experiences.

### Theme 4: my experiences give me a sense of purpose

Many participants felt that their experiences gave them a sense of purpose: that despite the trauma of their past experiences, their experience could be used for something good, and for some, this translated into career choices:

*‘I mean it has had a massive impact on my life … it has definitely shaped me. I guess to want to do something with that … to try and make change for others.’* (P02, IPV)

*‘Not that it makes up for what I went through, but it makes me feel like there’s purpose I guess in what I’ve been through.’* (P04, CA and abuse by parents as adult)

For some participants, their sense of purpose guided their career choice with many choosing medicine or general practice so that they could help others:

*‘It defined my choice of career. I really wanted to be a healer. I really wanted to offer space and kindness to people and I saw medicine as a way of doing that.’* (P01, CA and IPV)

Some even felt that working with survivors helped them in their own journey of healing:

*‘I think that being able to provide that support actually in some strange way, helps you as well.’* (P15, CA and IPV)

*‘One of the reasons I do this work is obviously because I want families to be healthy … and so doing work professionally which helps support that is actually quite enriching.’* (P02, CA)

For one participant, working with survivors prompted reflection about her own situation.

*‘Looking after patients going through that, while you’re going through it yourself … there were times where I was telling patients things that in my head … I should be telling this stuff to myself.’* (P15, CA and IPV)

Still, some participants had to put boundaries around DFV work with patients to protect themselves from becoming ‘burnt out’. This included choosing not to work rurally (P12), not to do paediatrics (P17, P14), working in a GP subspecialty (P10), and avoiding taking on a DFV education role (P04). However, these boundaries did not prevent them from going above and beyond for survivor patients they did identify.

In contrast, five participants had taken, or were planning to take, their sense of purpose a step further by advocating for survivors beyond the clinic in multiple ways: supporting doctor survivors; in advocacy work; research; education; and entering politics.

*‘But I do feel like I want to get into some sort of advocacy kind of work, because the system is really not good.’* (P15, CA and IPV)

## Discussion

### Summary

This is the first, to the authors’ knowledge, qualitative article to explore GP DFV survivors’ experiences when working with DFV survivor patients. Four themes were identified: my experiences can be my superpower (with subthemes of having a sixth sense and going above and beyond); connecting with survivors’ stories; wearing a cloak; and my experiences give me a sense of purpose. This research suggests there are both positive and negative impacts within the intersecting zone of being a survivor GP who is working with patients who are also DFV survivors.

### Strengths and limitations

Strengths of the study included that participants were from diverse locations throughout Australia and had a range of DFV experiences. Limitations included that all participants were women, despite trying to engage male GPs, which may limit transferability. Participants may have been more likely to be interested in the topic and may have been more likely to be advocates of DFV survivors leading them to take part in this study.^24^ This may be both a limitation and a strength as we potentially elevate the voices of DFV advocates. Participants may also have felt some level of distress or discomfort with this topic, which may have influenced their responses; however, it is likely that participants in sensitive-topic research experience positive outcomes from taking part.^25^

### Comparison with existing literature

The first theme identified, my experiences can be my superpower, related to participants perceiving that they were identifying and working with more survivor patients than their peers and offering them better care. This is similar to other literature including findings of a meta-analysis and systematic review of experiences of health professionals who had been subjected to DFV.^6,8,10,13,26–28^ Our study goes further by exploring why survivor GPs might provide better care to survivors. First, the survivors in this study had an intuitive sense of DFV because of their deep understanding of the patterns of behaviour underlying DFV. Second, they identified with survivors, which increased their empathy and gave them a powerful and unique understanding of their survivor patient’s situation.

However, the theme of connecting with patient stories conveyed that participants commonly experienced difficult emotions, especially, but not exclusively when they identified with patient stories. Clinicians who have experienced trauma have been shown to be at increased risk of experiencing secondary traumatisation when working with patients subjected to trauma and much of the literature focuses on the negative impact of this work on clinicians.^29–31^ However, a systematic review in mental health workers with a personal history of trauma found that although there was an association with poorer mental health, it was not uniformly related to worse outcomes.^29^ Although no previous studies, to the authors’ knowledge, have looked at the effect of being a GP survivor when hearing the stories of DFV survivor patients, a 2025 study of GPs found that encountering impactful patient stories often felt difficult, yet GPs found a source of meaning in them.^32^ Further study of support for GP survivors is recommended, particularly because of their perceived large DFV caseload.

The third theme of wearing a cloak is where there was tension in the consultation between the professional role and self-disclosure of stories with survivor patients. Self-disclosure, which is the disclosure of personal information by a clinician to their patient, remains controversial.^33^ A 2015 systematic review of nine studies of self-disclosure in primary care found that self-disclosure was common, although no studies looked at patient outcomes.^34^ Self-disclosure may help to build rapport, show empathy, increase patient disclosure, and increase trust.^33,35–37^ The patient–practitioner relationship has been described through the relationship-centred care model, which through humanising the professional relationship may improve health outcomes.^38^ However self-disclosure could be unhelpful for patients as it risks removing the focus from them, and could lead to unhealthy relationships through boundary violations and increased clinician’s anxiety.^33,35–37,39^ It is recommended that self-disclosure only occurs when clinicians know their patients well, have supporting patients as the goal, and should be used with caution.^37,40^ Participants in our study who shared their stories or cried with patients, said they were careful to only ever share relevant parts of their stories and they did this to support their survivor patients, however, there remained a tension in their professional role.

Despite this tension and the risk of secondary traumatisation, many participants felt that their experiences gave them a sense of purpose (theme 4). This aligns with one of the findings of a qualitative meta-synthesis where practitioners who had a ‘commitment’, including through personal experience of DFV, indicated greater readiness to do the work with survivors.^41^ A study of Australian clinicians demonstrated that survivor clinicians appeared to be doing more of the work seen as better clinical care of survivor patients.^13^ In our study, many survivor GPs based their career choice on their past experiences of DFV. This aligns with a systematic review that found that often ‘helping professionals’ are motivated to enter their occupation as a result of their experiences of abuse.^42^

### Implications for research and practice

General practice clinics need to understand that they will have medical staff who experience DFV and that rather than being a liability this might be a strength in responding to DFV in the patient population. A major implication is that support systems could be put in place for staff throughout their journey through DFV to alleviate any distress. Although GP DFV survivors commonly experienced difficult emotions, especially when they identified with survivor patients, they had a commitment to their survivor patients that stemmed from their own experiences of DFV. These experiences increased their empathy for these patients and gave them a deep understanding of their patients’ situations that allowed them to powerfully work together with their patients aiming to improve their situations. Further research is recommended into whether and how health care of survivor patients is affected when they are being managed by a GP survivor and how best to support GP survivors who may be detecting and supporting more DFV patients than their colleagues, acknowledging the risk of secondary traumatisation.
